# Effects of protease enzyme supplementation and varying levels of amino acid inclusion on productive performance, egg quality, and amino acid digestibility in laying hens from 30 to 50 weeks of age

**DOI:** 10.1016/j.psj.2022.102465

**Published:** 2022-12-31

**Authors:** I. Poudel, V.R. Hodge, K.G.S. Wamsley, K.D. Roberson, P.A. Adhikari

**Affiliations:** ⁎ Department of Poultry Science, Mississippi State University, Mississippi State, MS 39762 USA; † CSA Animal Nutrition, Dayton, OH 45414 USA

**Keywords:** digestibility, laying hen, low dAA/CP, phytase, protease

## Abstract

An experiment was conducted to evaluate the effects of protease supplementation and reduced digestible amino acid (**dAA**)/ crude protein (**CP**) level on productive performance, AA digestibility, and egg quality parameters in Hy-Line W-36 laying hen from 30 to 50 wk of age. A total of 768 hens (12 replicates of 8 hens per treatment) were equally and randomly allocated into 8 experimental diets in a 4 × 2 factorial arrangement of dAA/CP level (100, 95, 90, and 85% of breeder recommendation) and protease (exclusion or inclusion). Protease was added at 60 g/metric ton of feed in the inclusion group. Hens were housed in raised-wire cages with a stocking density of 870 cm^2^/bird. The adequate (100%) diet was based on corn and soybean meal and formulated based on the digestible (**d**) Lys and dAAs (dMet, dThr, dTrp, dTSAA, dIle, and dVal) to meet 100% of the current management guide recommendation. Variations in dAA/CP (95, 90, and 85% diets) were accomplished by reducing the 100% dAA by 5, 10, and 15%, respectively. All diets were supplemented with phytase at 500 phytase units (**FTU**)/kg. Data were analyzed using PROC GLM of SAS 9.4. There was a main effect of dAA/CP level on 85% diet where it had a lower mean hen-day egg production (**HDEP**, *P* < 0.01), egg mass (**EM**, *P* < 0.01), and higher feed conversion ratio (FCR, *P* < 0.001). Higher egg weight (*P* < 0.01) was observed in 95 and 100% dAA/CP level diets. However, Haugh unit (*P* < 0.01) and albumen height (*P* < 0.01) were higher in 85 and 90% diets. The inclusion of protease reduced the feed consumption (*P* = 0.0247), FCR for dozens of eggs (*P* = 0.0049) from 30 to 49 wk of age without affecting the HDEP or EM. Protease supplementation and dAA/CP level had an effect on the apparent ileal digestibility (**AID)** of CP (*P* = 0.019), Lys (*P* < 0.01), Thr (*P* < 0.01), Trp (*P* = 0.017), and Val (*P* < 0.01). Addition of protease significantly increased egg income (*P* = 0.033) and return on investment (*P* = 0.00223) from 30 to 37 wk of age. At 38 to 50 wk of age, dAA/CP level had a significant effect on egg income (*P* < 0.001), feed cost (*P* < 0.001), and return on investment (*P* < 0.001). This experiment indicates that the inclusion of protease in 90 and 95% lower dAA/CP diets could help improve the digestibility of CP, and key amino acids and maintain productive performance of corn and soybean meal-based diets in Hy-Line W-36 laying hen from 30 to 50 wk of age.

## INTRODUCTION

Feed cost accounts for approximately 60 to 70% of total production cost in the egg industry (Wilkinson, 2018). Out of this, 85% correlates to the energy and protein content of the laying hen diet (Santana et al., 2018). While reducing crude protein (**CP**) levels in diets may offer some advantages for the environment and reduce the feed cost, maintaining the birds' digestive health and development performance is difficult. With the addition of synthetic amino acids (**AA**) in the diet, it is now feasible to feed low CP diets by without compromising the birds’ digestive health and performance (Dong et al., 2017) and maintain egg production rates (Ji et al., 2014) with favorable economic (Burley et al., 2013) and environmental effects (Latshaw and Zhao, 2011; Alagawany et al., 2018). Furthermore, a wide range of exogenous enzymes can be supplemented in commercial layer diets to improve the digestibility of nutrients, reduce feed cost, and lower the environmental pollution from unused nutrients (Choct, 2006; Deniz et al., 2013; Ravindran, 2013; Geraert and Dalibard, 2015).

Previously, researchers have mainly focused on phytase (Van Der Klis et al., 1997; Liu et al., 2008), xylanase (Whiting et al., 2019), soy-based alpha-galactosidase (Llamas-Moya et al., 2021) and beta-glucanase (Karunaratne et al., 2022) in wheat (Gutierrez Del Alamo et al., 2008), and barley-based diets (Karunaratne et al., 2022). Previously endogenous proteases produced in the gastrointestinal tract (**GIT**) were widely thought to be adequate for complete feed protein utilization (Nir et al., 1993). However, evidence on poultry CP digestibility suggests that significant quantities of protein pass through the GIT without being fully digested (Lemme et al., 2004). Because of this undigested protein, additional exogenous proteases can be used in laying hen diets to increase protein digestibility. Exogenous protease supplementation in laying hen has shown to maintain production rates in nutritionally deficient diets (Barbosa et al., 2020) and even decrease feed conversion ratio in nutritionally adequate diets (Chen et al., 2021). Proteases generated by microorganisms are categorized according to whether they are acidic or basic. Functional groups and the location of the peptide bond are used further to categorize them (Razzaq et al., 2019). There are about 44 different types of mono-component proteases studied in 67 previous researches in poultry feed (Lee et al., 2018). It has been found that the addition of protease as a mono-component enzyme increases the pre-cecal digestibility of almost all AA in broilers (Angel et al., 2011; Fru-nji et al., 2011; Ding et al., 2016; Stefanello et al., 2016; Erdaw et al., 2017a; Cowieson et al., 2018), turkey (Vieira et al., 2013), and duck diets (Jiang et al., 2020; Wang et al., 2020b). Furthermore, when fed with phytase (Vieira et al., 2016; Jiang et al., 2020) protease has shown an increase in AA digestibility and growth performance and xylanase (Freitas et al., 2011). However, other studies that have used mono-component protease in combination with other enzymes have found no effect of protease supplementation on pre-cecal AA digestibility in broilers (Gutierrez Del Alamo et al., 2008; Kaczmarek et al., 2014; Rada et al., 2016; Borda-Molina et al., 2019; Walk et al., 2019). The divergent results in broiler studies with respect to pre-cecal AA digestibility could be due to differences in the experimental diet composition, supplementation level, or concomitant supplementation of other enzymes. Furthermore, there is increasing evidence that protease supplementation helps maintain egg production even in low CP diets (Barbosa et al., 2020). As such, literature has demonstrated that the limiting essential digestible amino acid (**dAA**) in corn and soybean meal (**SBM**) based diets; example: methionine (**Met**), lysine (**Lys**), threonine (**Thr**), tryptophan (**Trp**), valine (**Val**), Isoleucine (**Ile**), and total suphur AA (**TSAA**); can be elevated by enhancing the protein turnover into smaller polypeptides by the addition of exogenous protease enzymes (Bregendahl et al., 2008; López-Otín and Bond, 2008; Macelline et al., 2021). However, there is minimal research showing the effect of protease enzyme supplementation on productive performance and egg quality in laying hens. Furthermore, incorporating exogenous protease supplementation in low dAA/CP diets to maintain production performance and AA digestibility is not well studied. Therefore, we hypothesized that adding a commercially available protease enzyme to varying levels of dAA/CP of corn and SBM-based commercial laying hen diets will maintain the productive performance and improve the digestibility of major AAs. The objective of this study was to evaluate the effects of the mono-component protease supplementation in low dAA/CP level diets on various parameters such as egg production, performance, egg quality, and AA/CP digestibility in Hy-Line W-36 laying hen from 30 to 50 wk of age.

## MATERIALS AND METHODS

All animal care procedures were approved by the Mississippi State University Institution of Animal Care and Use Committee (Protocol number: IACUC-19-36).

### Pre-experimental Period

One-thousand-day-old Hy-Line W-36 pullet chicks were obtained from Hy-Line hatchery (Hy-Line North America, GA). Birds were reared in pullet cages with feed and water provided ad libitum. Feed was provided as starter 1, starter 2, grower, developer, and pre-lay as per Hy-Line guidelines (Hy-Line International, 2020). At 17 wk of age, birds were transferred to conventional A-frame cages (Big Dutchman, Calveslage, Germany), and from 18 wk of age, birds were switched to a peaking diet as per the management guide. At 28 wk of age, hens were randomly assigned to treatments and provided a 2-wk adaptational period to the experimental diets. Two adjacent cages with four birds per cage were fed in a standard feeder (116 cm long × 12.7 cm high × 11.4 cm wide) and were assigned as one experimental unit with a stocking density of 870 cm^2^/bird. Feed was distributed equally in all the feeders, and egg production was recorded once daily. If the cage was lower than the flock average regarding egg production, the birds were further separated into individual cages, and birds not laying were excluded from the trial.

### Experiment Design, Birds, and Feed

The experiment design was a randomized complete block design with location within the house as blocking criteria. A 4 × 2 factorial arrangement of dAA/CP levels (85, 90, 95, and 100%) and inclusion or exclusion of protease supplementation. Protease was added at 60 g/metric ton of feed in the inclusion group. The protease used in this study was a commercial enzyme produced by submerged fermentation of *Bacillus licheniformis* containing transcribed genes from *Nocardiopsis prasina*. Enzyme activity for this protease was measured in PROT units, with 1 unit defined as the amount of enzyme that releases 1 µmol of p-nitroaniline from 1 µM of substrate (Suc-Ala-Ala-Pro-Phe-p-nitroaniline) per minute at pH 9.0 and 37°C (DSM Nutritional Products AG, Kaiseraugst, Switzerland). In principle, the protease cleaves the substrate Suc-Ala-Ala-Pro-Phe-pNA releasing the chromogen para-nitroaniline (**pNA**). The amount of released yellow pNA is proportional to the protease activity of the enzyme and was measured photometrically at a wavelength of 405 nm. The method 101 SOY/02E was used and is a method developed by DSM (DSM Nutritional Products AG, Kaiseraugst, Switzerland). The enzyme was added to the feed by first mixing it with 1 oz of ground corn and then with the premix ingredients. All treatment diets were supplemented with a standard dose of 500 phytase units (FTU)/kg of Thermo protection Technology (TPT) coated phytase enzyme (Axtra PHY; Dupont Nutrition and Biosciences, Cedar Rapids, IA). The protease and phytase enzyme analysis of the experimental diets was completed by DSM Nutritional Products, Belvidere NJ. Both Ca and P were credited as 0.15% for the phytase used. A total of 768 Hy-Line W-36 laying hens were randomly allocated into one of 8 dietary treatments with 12 replicates each. The two cages (116 cm long × 60 cm wide) that had 8 birds shared a common feeder and were considered experimental units. The stocking density of hens at the beginning of the study was 870 cm^2^/bird. Throughout the study, water was provided ad libitum. The light was provided at the recommended hours (16L:8D) throughout the experimental period. Lights were switched on at 5:30 AM and switched off at 9:30 PM. All diets were fed in mash form and added at the rate of ∼100 g/bird/day. All nutrients were based on this feed intake rate to meet or exceed the requirement. According to the management guide, the hens were phase-fed to where layer 1 diets were provided from wk 30 to 37 and layer 2 diets from wk 38 to 50. The feed formulation and the determined analysis for layer 1 and layer 2 diets are shown in Table 1.

**Table 1:** Nutrient composition experimental diets (Layer 1 and Layer 2).

Ingredient (%)	Layer 1				Layer 2			
	100%	95%	90%	85%	100%	95%	90%	85%
Corn, Grain	56.24	58.75	62.00	63.05	57.52	59.05	61.68	64.80
Soybean meal, 48%	24.20	22.53	20.85	18.18	21.05	17.88	16.05	14.59
Distillers Grain/Sol	3.30	2.47	1.20	2.90	5.50	7.48	7.08	5.93
Limestone^1^	9.48	9.51	9.52	9.53	9.43	9.45	9.47	9.48
Poultry Fat	4.50	4.50	4.20	4.13	4.45	4.08	3.65	3.16
Dicalcium Phosphate	1.35	1.34	1.34	1.36	1.22	1.21	1.22	1.22
Salt	0.30	0.30	0.30	0.30	0.30	0.30	0.30	0.30
Vitamin Premix^2^	0.25	0.25	0.25	0.25	0.25	0.25	0.25	0.25
*DL*-Methionine	0.190	0.179	0.170	0.150	0.151	0.138	0.125	0.120
*L*-Lysine	0.075	0.080	0.080	0.077	0.080	0.110	0.121	0.120
Valine	0.050	0.035	0.023	0.020	0.000	0.000	0.000	0.000
Threonine	0.030	0.025	0.030	0.020	0.010	0.015	0.020	0.004
Phytase^3^	0.020	0.020	0.020	0.020	0.020	0.020	0.020	0.020
Protease^4^								
**Calculated values**^5^								
ME (Kcal/kg)	2880	2880	2880	2880	2850	2850	2850	2850
CP	17.58	16.70	15.82	14.94	16.30	15.49	14.67	13.86
Digestible Lys	0.84	0.80	0.76	0.71	0.77	0.73	0.69	0.65
Digestible Met	0.44	0.42	0.40	0.37	0.39	0.37	0.35	0.33
Digestible Thr	0.59	0.56	0.53	0.50	0.54	0.51	0.49	0.46
Digestible Try	0.18	0.17	0.16	0.15	0.16	0.15	0.14	0.14
Digestible Ile	0.67	0.64	0.60	0.57	0.62	0.59	0.56	0.53
Digestible Val	0.74	0.70	0.67	0.63	0.68	0.65	0.61	0.58
Calcium	4.00	4.00	4.00	4.00	3.95	3.95	3.95	3.95
Phosphorus (available)	0.45	0.45	0.45	0.45	0.40	0.40	0.40	0.40

1 Limestone was added as 50% fine and 50% coarse limestone in the experimental diet.

2 Vitamin A, 3,090,000 IU/g; Vitamin D3, 1,100,000 IU/kg; Vitamin E, 6200 IU/kg; Vitamin B1, 4.4 mg/kg; menadione, 330 mg/kg; riboflavin, 2,600 mg/kg, D-pantothenic acid, 2600 mg/kg; niacin, 11,000 mg/kg, choline, 150,000 mg/kg; folic acid, 275 mg/kg; pyridoxine, 550 mg/kg; thiamine, 440 mg/kg; biotin 13.2 mg/kg; Manganese, 40 g/kg; Zinc, 40 g/kg;

3 Phytase Dupont, AxtraPHY 500 TPT. The phytase in the experimental diets were analyzed by DSM Nutrional Products, Belvidere, NJ. The phytase recovery was above 90% from all the experimental diets. Both Ca and P were credited as 0.15% for the phytase used.

4 Protease included at 60 g/ metric ton in the expense of corn. The protease in the experimental diets were analyzed by DSM Nutrional Products, Belvidere, NJ. The protease recovery was 98% from the experimental diets with protease.

5 Values represented in g/bird/day.

### Egg Production and Performance

Throughout the study, egg production and downgraded eggs were recorded daily. Eggs were collected daily at 3:30 PM, within a half-hour window. To calculate hen-day egg production (**HDEP**), the total number of eggs produced by the experimental unit was divided by the total number of hen-days. Eggs were downgraded if they were anything less than Grade A according to the USDA grading system based on external attributes (USDA 2000). Egg mass (**EM**) was calculated weekly using the average egg weight (measured twice weekly) and multiplying it by the HDEP for that week. Each experimental unit had individual containers for feed storage. Feed intake was calculated every week by subtracting the amount of feed remaining in the feeders from the amount of feed added to the feeders that week. Feed conversion ratio per kg egg mass (**FCR-G**) was calculated by feed intake for that week divided by EM for that week.

$$\text{FCR}-G\;(\text{per}\;\text{kg}\;\text{egg}\;\text{mass})=\frac{\text{Kg}\;\text{of}\;\text{feed}\;\text{consumed}\;\text{that}\;\text{week}}{\text{EM}\;\text{that}\;\text{week}}$$

Similarly, the feed conversion ratio per dozen eggs (**FCR-D**) was calculated by multiplying feed intake by 12 and dividing by total egg produced for that week. Body weight was measured at the beginning of the study and at the end of each diet change.

$$\text{FCR}-D\;(\text{per}\;\text{dozen}\;\text{eggs})=\frac{\text{Kg}\;\text{of}\;\text{feed}\;\text{consumed}\;\text{that}\;\text{week}\;\times \;12}{\text{Total}\;\text{eggs}\;\text{produced}\;\text{that}\;\text{week}}$$

#### Egg Parameters

A total of 288 eggs (3 from each experimental unit × 96 experimental units) were collected randomly every 5 wk to determine egg weight and assess egg quality and eggshell quality. Abnormal eggs such as pee wee, soft-shelled, cracked, and double-yolked eggs were excluded during the evaluation of the egg quality. Egg quality analysis included egg weight (**EW**), specific gravity (**SG**), albumen height (**AH**), Haugh Unit (**HU**), yolk weight (**YW**), yolk percentage (**YP**), and albumen percentage (**ALB**). Eggshell quality included shell weight (**SW**), shell percentage (**SP**), and shell thickness (**ST**). The SG for each was determined through submersion in saltwater solutions with densities ranging from 1.060 to 1.095 at 0.005 intervals, utilizing the methods described by Peebles and McDaniel (2013). Egg weight was measured using a digital balance; immediately after, Haugh units were calculated by breaking a fresh egg on a level flat surface and then calculating AH by using the TSS QCD apparatus (Technical Services and Supplies Ltd, York, UK). To obtain the weight of the yolk, chalaza was removed from the yolk using forceps, and the yolk was rolled on a dry paper towel to remove any adhering albumen. The albumen weight was determined by subtracting yolk and shell weights from the total egg weight. After breaking eggs for internal egg quality, the eggshells were collected and washed with clean water to remove all the adhering albumen. The cleaned eggshells were dried at room temperature for 2 consecutive days, and the eggshell weight and thickness were recorded with intact shell membranes. To calculate the percent shell weight, SW was divided by EW and multiplied by 100. Eggshell thickness was measured at 3 different sites along the equator, the narrow end and the broad end using an Ames micrometer (B.C. Ames Incorporated, Framingham, MA); the average reading of the three measurements was recorded and used for analysis.

### Apparent Ileal Digestibility

At 50 wk of age, birds of the same treatment were fed a standard diet containing 0.5% of an indigestible feed marker (TiO2) for 5 d to determine CP and dAA digestibility. One bird from each experimental unit was euthanized and the ileal content was collected from the distal end of the ileum extending from 3 cm below the Meckel's diverticulum to 3 cm proximal to the ileocecal junction. The digesta were flushed with distilled water, frozen, and freeze-dried in preparation for the CP, dAA, and TiO_2_ recovery. The digesta were then analyzed for TiO_2_, crude protein and dAA content (A.T.C Scientific, North Little Rock, AR). The analysis was carried out in duplicate, and results were reported on a DM basis. The following equation was used to calculate apparent ileal digestibility (Lemme et al., 2004).

$$\text{AID}\;\left(\%\right)=\frac{{\left(\frac{\text{AA}}{\text{Ti}O_{2}}\right)}_{\text{Diet}}-\;{\left(\frac{\text{AA}}{\text{Ti}O_{2}}\right)}_{\text{Ileum}\;}\;}{{\left(\frac{\text{AA}}{\text{Ti}O_{2}}\right)}_{\text{Diet}}}\times \;100$$

### Economic Analysis

Return on investment was calculated using egg income and feed cost for 30 to 37 wk of age and 38 to 50 wk of age. To estimate the egg income, the average eggs weight from each experimental unit was categorized in 3 different categories every 2 wk. The Southeastern USDA prices for extra-large, large, and medium eggs were used for income tabulation in dollars per dozen for that 2-wk period. The prices for extra-large, large, and medium eggs were $2.45, $2.23, $1.84 per dozen of eggs at the time of data analysis. Feed costs were calculated by multiplying the local ingredient prices at the time of analysis and by feed intake per hen during that period. Moreover, feed costs were subtracted from egg income to find a return per dozen eggs.

#### Statistical Analysis

Data were tested for normality by the PROC UNIVARIATE procedure. All analyzed data were considered sufficiently normally distributed based on the residuals' graphical evaluation (QQ plot). A 2-way ANOVA was used using the PROC Generalized Linear Model (**GLM**) procedure of SAS 9.4 (2019) (SAS Inc., Cary, NC). Treatment, hen age, and blocks were considered fixed effects. Treatments were analyzed as a 4 × 2 factorial arrangement of varying dAA/CP (100, 95, 90, and 85%) and inclusion of protease (with or without). Significant main effects or interactions were separated using Tukey's HSD test. Statistical significance was defined at *P*-value less than or equal to 0.05.

## RESULTS

### Egg Production and Performance Data

The dietary treatments were analyzed for their nutrient values as shown in Table 2. Diets were also tested for enzyme recovery, with more than 90% enzyme recovery for phytase and more than 98% recovery for the protease enzyme in the mixed feed.

**Table 2:** Analyzed nutrient composition of the experiment diets (layer 1 and 2).

CP^1^/AA^2^	Layer 1								Layer 2							
	Without protease				With protease				Without protease				With protease			
	100%	95%	90%	85%	100%	95%	90%	85%	100%	95%	90%	85%	100%	95%	90%	85%
CP	17.54	16.73	15.93	14.12	17.35	16.76	15.82	14.84	16.91	16.96	15.35	13.82	16.93	16.26	14.58	13.83
Lys	0.96	0.85	0.78	0.74	0.90	0.90	0.82	0.72	0.85	0.81	0.84	0.72	0.88	0.88	0.73	0.67
Met	0.41	0.41	0.39	0.37	0.44	0.40	0.38	0.36	0.40	0.38	0.39	0.33	0.38	0.35	0.36	0.34
Cys	0.33	0.34	0.35	0.31	0.34	0.34	0.32	0.31	0.32	0.32	0.31	0.26	0.30	0.31	0.28	0.29
Thr	0.67	0.60	0.55	0.54	0.65	0.56	0.53	0.56	0.61	0.55	0.57	0.50	0.61	0.60	0.54	0.49
Try	0.20	0.19	0.16	0.17	0.19	0.17	0.18	0.16	0.19	0.18	0.17	0.18	0.18	0.18	0.16	0.14
Ile	0.68	0.61	0.54	0.52	0.65	0.59	0.54	0.58	0.64	0.62	0.59	0.50	0.64	0.62	0.54	0.51
Val	0.77	0.68	0.63	0.60	0.73	0.67	0.61	0.65	0.70	0.67	0.67	0.57	0.71	0.69	0.67	0.59

All samples were analyzed by ATC scientific, North Little Rock, AR.

1 Analyzed using AOAC 968/06-990.03.

2 Analyzed using AOAC 994.12/988.15/982.30.

Throughout the experiment, significance for the main effect of dAA/CP level on HDEP (*P* < 0.0001), EM (*P* < 0.0001), and EW (*P* < 0.0001) were observed, as shown in Table 3. Overall, higher HDEP of 91.90, 91.91, and 90.43% was observed in 100, 95, and 90% dAA/CP groups, respectively, compared to 86.23% production in the 85% dAA/CP level group from 30 to 49 wk of age (Table 2). Similarly, significantly lower EM was observed in the 85% group (50.25 g) compared to 90% (53.10 g); both of which were lower than 95% and 100% dAA/CP groups (54.74 and 54.51 g, respectively). Similarly, a lower egg weight of 58.32 g was observed at 85% dAA/CP level as compared to 59.55 and 59.29 g at 95 and 100% dAA/CP levels, respectively. The main effect of protease on HDEP was observed at 34 to 37 wk of age (*P* = 0.0125), where hens fed diets with protease had higher HDEP (94.48%) than those without protease (93.28%). Similarly, the main effect of protease inclusion was observed for EM at 34 to 37 wk of age (*P* = 0.0370). For this week range, the diets with protease had a significantly higher EM (56.49 g) than those without protease (55.72 g). At 46 to 49 wk of age, we observed an interaction of dAA/CP levels and protease inclusion on EM (*P* = 0.0386) where, inclusion of protease at lower dAA/CP level (85% and 90%) had beneficial effect on EM as compared to higher dAA CP level (95% and 100%).

**Table 3:** Effects of varying dAA/CP levels and protease supplementation on egg production and egg mass.

dAA/CP Level	Protease	30–33 wk			34–37 wk			38–41 wk			42–45 wk			46–49 wk			30–49 wk		
		HDEP^1^	EM^2^	EW^3^	HDEP	EM	EW	HDEP	EM	EW	HDEP	EM	EW	HDEP	EM	EW	HDEP	EM	EW
85%	-	92.86	54.77	58.87	90.72	53.79	58.61	83.58	48.13	57.57	80.32	46.50	57.82	70.74	41.04^c^	58.00	85.56	49.82	58.23
	+	92.69	54.16	58.46	93.75	55.16	58.77	86.45	49.14	56.35	79.47	46.57	58.72	73.71	45.17^bc^	61.42	86.90	50.68	58.42
90%	-	95.34	56.13	59.13	93.56	55.51	59.01	87.36	50.51	57.81	88.47	52.14	58.92	80.44	46.71^ab^	58.07	90.07	52.82	58.61
	+	95.89	56.65	59.50	94.53	56.42	59.70	87.97	51.06	58.09	89.67	50.53	56.48	81.18	48.23^ab^	59.45	90.79	53.37	58.81
95%	-	95.07	57.23	59.53	94.25	56.92	60.82	91.49	53.35	58.30	90.35	53.54	59.23	84.88	51.21^a^	60.33	92.23	54.99	59.60
	+	95.66	56.68	59.63	95.21	57.06	60.14	89.78	52.55	58.55	89.52	53.55	59.88	84.07	50.03^ab^	59.52	91.23	54.48	59.50
100%	-	96.13	57.05	59.35	96.06	57.97	60.37	90.18	52.42	58.13	87.03	51.90	59.65	82.62	49.40^ab^	59.78	91.60	54.40	59.39
	+	96.38	57.26	59.40	96.04	57.96	60.57	91.94	53.81	58.53	87.94	52.20	59.33	81.55	48.66^ab^	57.33	92.19	54.61	59.21
SEM		0.604	0.462	0.316	0.580	0.417	0.393	0.872	0.641	0.457	1.204	1.223	1.145	1.376	1.251	1.324	0.660	0.469	0.288
Main effect																			
85%		92.77^b^	54.46^b^	58.67^b^	91.59^b^	53.73^c^	58.69^b^	84.51^c^	48.13^c^	56.96^b^	79.89^b^	46.54^b^	58.27	72.25^c^	43.10	59.71	86.23^b^	50.25^c^	58.32^c^
90%		95.08^a^	56.38^a^	59.31^ab^	94.05^a^	55.82^b^	59.35^b^	87.66^b^	50.79^b^	57.95^ab^	89.07^a^	51.34^a^	57.70	80.81^b^	47.47	58.77	90.43^a^	53.10^b^	58.71^bc^
95%		95.48^a^	56.95^a^	59.58^a^	94.38^a^	57.07^ab^	60.48^a^	90.63^a^	52.95^a^	58.43^a^	89.94^a^	53.55^a^	59.55	84.48^a^	50.62	59.93	91.91^a^	54.74^a^	59.55^a^
100%		96.25^a^	57.15^a^	59.38^ab^	95.50^a^	57.79^a^	60.47^a^	91.06^a^	53.11^a^	58.33^a^	87.49^a^	52.05^a^	59.49	82.08^ab^	48.03	58.56	91.90^a^	54.51^a^	59.29^ab^
SEM		0.427	0.333	0.223	0.476	0.363	0.281	0.574	0.459	0.323	0.611	0.865	0.810	0.681	0.885	0.936	0.399	0.331	0.203
																			
	-	94.94	56.30	59.22	93.28^b^	55.72^b^	59.70	88.15	51.10	57.95	86.55	51.02	58.90	80.13	47.09	59.05	89.87	53.01	58.96
	+	94.84	56.19	59.24	94.48^a^	56.49^a^	59.79	88.78	51.39	57.88	86.65	50.71	58.60	79.67	47.52	59.43	90.36	53.29	58.98
	SEM	0.302	0.233	0.160	0.287	0.258	0.198	0.436	0.325	0.228	0.432	0.611	0.572	0.688	0.625	0.662	0.330	0.234	0.144
Statistical probability																			
dAA/CP level		<0.0001	<0.0001	0.0293	<0.0001	<0.0001	<0.0001	<0.0001	<0.0001	0.0060	<0.0001	<0.0001	0.2856	<0.0001	<0.0001	0.6638	<0.001	<0.001	<0.001
Protease		0.8191	0.7487	0.9201	0.0125	0.0370	0.7391	0.3079	0.5285	0.8268	0.9029	0.7252	0.7121	0.6391	0.6265	0.6817	0.2886	0.4022	0.9002
dAA/CP level × Protease		0.7594	0.5401	0.6736	0.3957	0.3018	0.3773	0.1480	0.3817	0.2458	0.7393	0.8564	0.4573	0.4423	0.0386	0.1415	0.4938	0.5001	0.8723

a-d Means within columns with different superscripts are significantly different at *P* ≤ 0.05.

1 HDEP (%) = hen day egg production. The hen-day egg production was averaged for the given period.

2 EM (gram/hen/day) = egg mass was calculated weekly using the average egg weight, measured twice a week, and multiplied by HDEP for that week. EM was averaged for the time period.

3 EW (Egg weight) was calculated weekly using the average egg weight, measured twice a week recorded in grams.

Similarly, at 46 to 49 wk of age, an interaction was observed between dAA/CP and inclusion of protease for FI (*P* = 0.0305), where 85% dAA/CP had lower FI as compared to 95% and 100% without and with protease, respectively as shown in Table 4. Furthermore, the main effect of protease was observed in FI from 30 to 49 wk of age (*P* = 0.0247). A higher FI of 110 g per bird/day was observed in the group without protease compared to 108 g of feed per bird/day from 30 to 49 wk of age. The main effect of dAA/CP was observed in FCR-G (gram of feed per gram of egg) at 30 to 33 wk (*P* = 0.0390), 38 to 41 wk (*P* < 0.001), 42 to 45 wk (*P* < 0.001), and overall, from 30 to 49 wk (*P* < 0.001). In general, 85% dAA/CP had higher FCR-G than 95 and 100% dAA/CP for these weeks. Similarly, the main effect of dAA/CP was observed in FCR-D at 38 to 41 wk (*P* < 0.001), 42 to 45 wk (*P* < 0.001), 46 to 49 wk (*P* = 0.0030) and an overall effect from 30 to 49 wk (*P* < 0.001), indicating the 85% dAA/CP had higher FCR-D as compared to 90, 95, and 100% dAA/CP for these weeks

**Table 4:** Effects of varying dAA/CP levels and protease supplementation on feed intake (FI), feed conversion ratio gram of feed per gram of egg (FCR-G) and feed conversion ratio kilogram per dozen (FCR-D) from 30 to 49 wk of age.

dAA/CP Level	Protease	30–33 wk			34–37 wk			38–41 wk			42–45 wk			46–49 wk			30–49 wk		
		FI^1^	FCR-G^2^	FCR-D^3^	FI	FCR-G	FCR-D	FI	FCR-G	FCR-D	FI	FCR-G	FCR-D	FI	FCR-G	FCR-D	FI	FCR-G	FCR-D
85%	-	113	2.08	1.47	109	2.08	1.69	106	2.21	1.54	109	2.29	1.59^a^	101^c^	2.48	1.71	108	2.21	1.54
	+	113	2.09	1.46	107	1.98	1.36	106	2.24	1.50	112	2.35	1.64^a^	105^bc^	2.36	1.72	109	2.20	1.52
90%	-	118	2.11	1.51	111	2.01	1.43	105	2.08	1.43	108	2.05	1.45^b^	112^ab^	2.44	1.69	110	2.11	1.48
	+	114	2.01	1.44	106	1.89	1.35	104	2.05	1.43	106	2.08	1.43^b^	108^ab^	2.25	1.60	107	2.04	1.43
90%	-	113	1.98	1.43	108	1.93	1.41	105	1.97	1.38	106	1.97	1.41^b^	113^a^	2.22	1.62	109	1.99	1.43
	+	110	1.97	1.40	114	2.00	1.45	104	1.97	1.39	106	2.00	1.44^b^	109^ab^	2.19	1.57	108	2.01	1.43
100%	-	117	2.07	1.46	114	1.99	1.46	104	2.00	1.39	110	2.14	1.52^ab^	112^ab^	2.27	1.63	111	2.08	1.48
	+	114	2.00	1.42	109	1.87	1.36	103	1.93	1.35	105	2.00	1.40^b^	113^a^	2.48	1.68	108	2.02	1.42
SEM		2.1	0.041	0.029	2.3	0.048	0.087	1.4	0.040	0.025	1.7	0.053	0.029	1.6	0.081	0.031	0.7	0.026	0.015
Main effect																			
85%		113	2.08^a^	1.47	108	2.03	1.42	106	2.23^a^	1.52^a^	111	2.32^a^	1.61	103	2.42	1.71^a^	109	2.20^a^	1.53^a^
90%		116	2.06^ab^	1.47	108	1.95	1.39	104	2.07^a^	1.43^b^	107	2.07^b^	1.44	110	2.35	1.65^ab^	109	2.07^b^	1.46^b^
95%		112	1.97^b^	1.41	111	1.96	1.42	104	1.97^bc^	1.39^b^	106	1.98^b^	1.43	111	2.20	1.59^b^	108	2.00^c^	1.43^b^
100%		116	2.03^ab^	1.44	111	1.93	1.41	104	1.96^c^	1.37^b^	107	2.07^b^	1.46	112	2.38	1.65^ab^	110	2.05^bc^	1.45^b^
SEM		1.4	0.029	0.020	1.7	0.034	0.023	1.0	0.029	0.017	1.2	0.038	0.020	1.1	0.057	0.022	0.7	0.018	0.011
	-	115	2.06	1.47^a^	110	2.00	1.44^a^	105	2.07	1.43	108	2.11	1.49	110	2.35	1.66	110^a^	2.10	1.48^a^
	+	113	2.02	1.43^b^	109	1.94	1.38^b^	104	2.05	1.42	107	2.11	1.47	109	2.32	1.64	108^b^	2.07	1.45^b^
	SEM	1.0	0.020	0.014	1.2	0.024	0.016	0.7	0.020	0.012	0.8	0.027	0.014	0.8	0.041	0.016	0.5	0.013	0.008
Statistical probability																			
dAA/CP level		0.1690	0.0390	0.1474	0.2510	0.1757	0.7920	0.3191	<0.0001	<0.0001	0.0506	<0.0001	<0.0001	<0.0001	0.0598	0.0030	0.5284	<0.0001	<0.0001
Protease		0.1015	0.1578	0.0474	0.2705	0.0521	0.0144	0.3915	0.4599	0.3441	0.3112	0.8903	0.3348	0.5375	0.6130	0.3013	0.0247	0.0878	0.0049
dAA/CP level × Protease		0.7906	0.4516	0.7968	0.0942	0.1360	0.1202	0.9680	0.6426	0.6247	0.1146	0.2433	0.0175	0.0305	0.0754	0.1501	0.1023	0.3026	0.1444

a-d Means within columns with different superscripts are significantly different at P ≤ 0.05.

1 FI= Feed intake (g); gram of feed consumed per bird/day. Feed intake was calculated every two weeks. FI was averaged for the time mentioned above

2 FCR-G= Feed conversion ratio (Gram of feed for gram of egg produced).

3 FCR-D= Feed conversion ratio (Kilogram of feed per dozen of eggs produced)

### Egg Quality Parameters

A higher AH of 8.94 and 8.96 was observed in 85 and 90% compared to 8.69 and 8.75 in 95 and 100% dAA/CP levels (*P* = 0.0011). Similarly, we observed with HU (*P* < 0.001) where a higher value of 94.46 and 94.41 was observed in 85 and 90%, respectively, compared to 93.00 and 93.20 in 95 and 100% groups. An interaction of dAA/CP level and protease inclusion was observed on YP (*P* = 0.013), where 85% level and without protease had a lower YP compared to the other increasing dAA levels either with or without protease, as shown in Table 5. We did not observe any direct effect of protease on ALB, SP, and ST.

**Table 5:** Effects of varying dAA/CP levels and protease supplementation on egg quality from 35 to 50 wk of age.

dAA/CP Level	Protease	SG	AH	HU	YW	YP (%)	ALB (%)	SP (%)	ST
85%	-	1.148	8.96	94.37	15.49^c^	26.13^c^	69.35	9.43	38.12
	+	1.091	8.92	94.55	15.56^c^	26.64^ab^	68.90	9.11	37.66
90%	-	1.086	8.85	93.86	15.93abc	26.85^a^	68.88	9.04	38.13
	+	1.086	9.06	94.96	15.66^bc^	26.34^ab^	69.40	9.02	37.88
95%	-	1.086	8.64	92.71	16.12^ab^	26.68^ab^	69.03	8.93	37.87
	+	1.086	8.75	93.28	16.06^ab^	26.69^ab^	69.23	8.94	37.80
100%	-	1.086	8.81	93.56	15.80abc	26.26^ab^	69.37	8.99	37.92
	+	1.090	8.69	92.85	16.24^a^	26.80^ab^	69.26	8.87	37.48
SEM		0.0201	0.082	0.389	0.108	0.158	0.732	0.161	0.309
Main effect									
85%		1.119	8.94^a^	94.46 a	15.52	26.39	69.12	9.27	37.89
90%		1.086	8.96^a^	94.41 a	15.79	26.59	69.14	9.03	38.01
95%		1.086	8.69^b^	93.00^b^	16.09	26.69	69.13	8.93	37.84
100%		1.088	8.75^b^	93.20^b^	16.02	26.53	69.32	8.93	37.70
SEM		0.0143	0.057	0.277	0.077	0.112	0.516	0.115	0.218
									
	-	1.102	8.82	93.63	15.83	26.48	69.16	9.10	38.01
	+	1.088	8.85	93.91	15.88	26.62	69.20	8.98	37.71
	SEM	0.0101	0.040	0.196	0.054	0.079	0.366	0.081	0.154
Statistical probability									
dAA/CP level		0.2725	0.0011	<0.001	<0.001	0.5174	0.9922	0.1212	0.8028
Protease		0.3576	0.5081	0.3070	0.5463	0.4268	0.9367	0.3302	0.1620
dAA/CP level × Protease		0.3600	0.1792	0.1320	0.0093	0.0130	0.9210	0.7169	0.9141

a-c Means within columns with different superscripts are significantly different at *P* ≤ 0.05. Abbreviations: AH, albumen height (mm); ALB, albumen percent (%); HU, haugh unit; P, eggshell percentage (%); SEW, egg weight (g); SG, specific gravity; ST, eggshell thickness (mm); YW, yolk weight(g); YP, yolk percent (%).

### Apparent Ileal Digestibility

An interaction effect was observed for AID of protein (*P* = 0.0437; Table 6). Protease inclusion did not affect the AID of protein at 85 % CP levels. The digestibility of protein in diets without protease at 90 and 95% was 74.71 and 75.258%, respectively which was lower than the 100% group without protease (81.69%). An interaction effect was observed for AID of Lys (*P* = 0.0036) with CP level and protease inclusion (Table 6). Higher AID of Lys was observed in 95% (with protease) and 100% (without protease) as compared to 90% without protease. An interaction effect was also observed for Thr digestibility (*P* = 0.0005). A lower AID of Thr of 66.29 and 67.87% was observed in diets without protease at 90 and 95% CP levels compared to 74.00% in 95% CP level with protease. This trend is reversed at 100% CP level, where diet without protease had a higher AID of Thr of 75.19% as compared to 66.22% with protease. An interaction was observed for AID of Trp with dAA/CP level and protease inclusion (*P* = 0.0138). A lower AID of Trp was observed at 85% (with protease) and 90% (without protease) as compared to 100% (without protease). An interaction of protease and dAA/CP was observed with Val (*P* = 0.0002) where higher values were observed in 90% supplemented with protease.

**Table 6:** Effects of varying dAA/CP levels and protease supplementation on apparent ileal digestibility (%) of protein and amino acids at 50 wk of age.

.dAA/CP Level	Protease	Apparent ileal digestibility (%)						
		CP^1^	Lys^2^	Met^2^	Thr^2^	Trp^2^	Ile^2^	Val^2^
85%	-	78.23^ab^	80.30^ab^	89.25	70.20^ab^	71.93^ab^	80.62	78.11^ab^
	+	78.42^ab^	83.27^ab^	87.42	71.53^ab^	67.27^b^	81.74	78.79^ab^
90%	-	74.71^b^	77.99^b^	87.09	66.29^b^	66.65^b^	78.52	73.75^c^
	+	77.44^ab^	81.15^ab^	88.26	70.15^ab^	71.96^ab^	82.49	78.83^ab^
95%	-	75.25^b^	79.19^ab^	86.95	67.87^b^	70.32^ab^	81.55	75.04^bc^
	+	77.67^ab^	83.67^a^	88.67	74.00^a^	72.17^ab^	81.83	79.00^ab^
100%	-	81.69^a^	84.11^a^	89.96	75.19^a^	78.01^a^	79.74	81.30^a^
	+	77.14^ab^	79.78^ab^	87.95	69.22^ab^	72.79^ab^	78.56	75.39^bc^
SEM		1.321	1.175	1.117	1.294	1.698	2.018	1.133
Main effect								
85%		78.32	81.78	88.34	70.87	69.60	81.18	78.45
90%		76.08	79.57	87.68	68.22	69.31	80.51	76.29
95%		76.46	81.43	87.81	70.93	71.24	81.69	77.02
100%		79.42	81.95	88.95	72.21	75.40	79.15	78.34
SEM		0.932	0.829	0.788	0.913	1.198	1.423	0.800
								
	-	77.47	80.40	88.31	69.89	71.73	80.11	77.05
	+	77.67	81.97	88.07	71.23	71.05	81.16	78.00
	SEM	0.665	0.592	0.563	0.652	0.855	1.016	0.571
Statistical probability								
dAA/CP level		0.0756	0.2156	0.7023	0.0462	0.0066	0.653	0.2203
Protease		0.8392	0.0743	0.7714	0.1622	0.5835	0.4787	0.2555
dAA/CP level × Protease		0.0437	0.0036	0.2526	0.0005	0.0138	0.6764	0.0002

a-c Means within columns with different superscripts are significantly different at *P* ≤ 0.05.

1 Analyzed using AOAC 968/06-990.03.

2 Analyzed using AOAC 994.12/988.15/982.30.

### Economic Analysis

As shown in Table 7, from 30 to 37 wk of age the main effect of dAA/CP level (*P* < 0.001) and protease (*P* = 0.0331) was seen on egg income. The egg income increased as dAA/CP increased from 85% to 100%. Similarly, a higher income of $2.46 per hen/2 wk was observed in groups with protease as compared to $2.43 per hen/2 wk in groups without protease from 30 to 37 wk of age. A higher feed cost (*P* = 0.0022) of $1.1 per bird/ 2 wk was observed in 100% dAA/CP diet compared to $1.05 and $1.03 hen/ 2 wk in 90% and 85% dAA/CP level, respectively. The addition of protease significantly increased the return on investment from 30 to 37 wk of age (*P* = 0.0223). From 38 to 50 wk of age, dAA/CP level had a significant effect on egg income (*P* < 0.001), feed cost (P < 0.001), and return on investment (*P* < 0.001). It was observed that increasing the dAA/CP from 85% to 90% and then to 95% significantly increased egg income and return on investment. However, increasing from 95% to 100% did not result in increased egg income, feed cost, and return on investment from 38 to 50 wk of age.

**Table 7:** Effects of varying dAA/CP levels and protease supplementation on egg income, feed cost, and return on investment from 30 to 50 wk of age.

		30–37 wk of age^1^			38–50 wk of age^1^		
dAA/CP level	Protease	Egg income^2^	Feed cost^3^	Return^4^	Egg income^2^	Feed cost^3^	Return
85%	-	2.34	1.03	1.29	2.01	0.93	1.08
	+	2.40	1.02	1.37	2.04	0.95	1.09
90%	-	2.43	1.07	1.35	2.21	0.95	1.26
	+	2.46	1.03	1.42	2.24	0.94	1.29
95%	-	2.47	1.06	1.39	2.32	0.97	1.35
	+	2.47	1.08	1.38	2.29	0.96	1.33
100%	-	2.49	1.12	1.36	2.25	0.99	1.25
	+	2.49	1.09	1.40	2.31	0.97	1.34
SEM		0.018	0.020	0.027	0.024	0.009	0.026
Main effect							
85%		2.37^c^	1.03^b^	1.33	2.02^c^	0.94^c^	1.08^c^
90%		2.44^b^	1.05^b^	1.39	2.22^b^	0.95^bc^	1.27^b^
95%		2.47^ab^	1.07^ab^	1.39	2.30^a^	0.96^ab^	1.34^a^
100%		2.49^a^	1.10^a^	1.38	2.28^ab^	0.98^a^	1.29^ab^
SEM		0.013	0.014	0.019	0.017	0.006	0.018
	-	2.43^b^	1.07	1.35^b^	2.20	0.96	1.24
	+	2.46^a^	1.05	1.39^a^	2.22	0.96	1.26
	SEM	0.009	0.009	0.013	0.013	0.012	0.004
Statistical probability							
dAA/CP level		<0.0001	0.0022	0.1228	<0.0001	<0.0001	<0.0001
Protease		0.0331	0.2232	0.0223	0.2087	0.4349	0.1356
dAA/CP level × Protease		0.2365	0.5878	0.3547	0.2787	0.1158	0.2116

a-c Means within columns with different superscripts are significantly different at *P* ≤ 0.05.

1 Economical analysis was done as $/ per hen/ for 2 weeks at the time of analysis.

2 Egg income was calculated in using hen day egg production, average egg weight and price of egg for the two wk period. The USDA prices for extra-large, large, and medium eggs were $2.45, $2.23, and $1.83 per dozen at the time of analysis. Egg income was expressed as $/hen/2 weeks.

3 Feed cost was calculated bases on the feed intake and local price of the feed ingredient calculated in proportion to percentage mixed in the diet at the time of analysis. The cost of corn, SBM, Distillers grain, Limestone, Dicalcium phosphate, Salt and fat $11.30, $18.8, $15.00, $8.3, $30.4, $6.95, and $30.00 for 22.68 kg bag respectively. The cost of Lys, Met, Thr, Val, Ile, vitamin premix, phytase, and protease was $1.40, $2.55, $1.38, $5.50, $10.50, $2.13, $22.50, and $40.80 per kg, respectively. Feed cost was expressed as $/hen/2 weeks.

4 Return on investment was calculated by subtracting feed cost from egg income.

## DISCUSSION

The objective of this study was to determine the effects of protease on reduced levels of dAA/CP on productive performance, egg quality, and AA digestibility in Hy-Line W-36 laying hen from 30 to 50 wk of age. Our findings with reduced dAA/CP are similar to previous studies that have reported that decreasing dietary CP levels can have a negative effect on egg production, egg weight, and egg mass (Rama Rao et al., 2014; dePersio et al., 2015), and also egg solids, and profit (Wu et al., 2007). A decrease in egg weight was observed even at 90% dAA/CP level, whereas a difference in egg production was observed only in the most deficient diet (85% dAA/CP level). Previous research has found that laying hens can largely manage their feed intake to satisfy their energy needs for egg production, and the amount of CP taken determines the average egg weight (Antar et al., 2004). This is a possible explanation for why we observed a reduction in egg weight at 95% and a decrease in HDEP and EM in more deficient diets. We observed a constant FI throughout the treatments even with varying dAA/CP levels. Therefore, by meeting their energy requirement even in low dAA/CP level diets, we had a constant overall feed intake but showed a reduction in the egg size. It was expected that the addition of protease to corn and SBM-based laying hen diets might improve feed conversion, increase egg weight, and enhance egg composition (Jaroni et al., 1999; Khan et al., 2011; Filho et al., 2015; Vieira et al., 2016; Park et al., 2020; Chen et al., 2021). Vieira et al. (2016) reported that protease supplementation in deficient diets, at 95 to 97% dAA/CP, could maintain their EW, AH, and HU and reduce the FI in Hy-Line W-36 hens as compared to recommended diet in the peak production phase. This is supported by our results, which indicate that the addition of protease in 90 and 95% dAA/CP of the recommendation level-maintained egg production. The novel findings from this research are that reducing the dAA/CP level up to 90% does not affect the egg production but reduces egg size. However, reducing below 85% can negatively impact the egg production and egg weight even with protease supplementation. In addition, it we conclude that EW, YW, YP and dAA digestibility may be more sensitive indicators of protease activity than egg production. This is also supported by Wu et al. (2007), who observed that decreasing the nutrient density linearly decreases the egg weight with a 2.2% decrease in egg weight for 5% decrease in dAA/CP level. On the other hand, we also observed that protease supplementation maintains the YP. This contradicts Vieira et al. (2016), who observed that the EW YP, and AH were unaffected by protease. However, there is a lack of extensive research on the effect of protease on EW, HU, and YP, which makes it premature to make any conclusions.

The objective of reducing the dAA/CP levels was to observe if protease supplementation would improve dAA/CP digestibility in these low dAA/CP levels in corn and SBM-based diets. These speculations were based on previous studies that have shown that supplementation of exogenous protease in broiler and turkey diets increases the prececal digestibility of almost all AA (Fru-nji et al., 2011; Vieira et al., 2013; Stefanello et al., 2016; Cowieson et al., 2018; Bertechini et al., 2020). Furthermore, protease supplementation has been shown to enhance trypsin activity as well as CP digestibility (Freitas et al., 2011; Ding et al., 2016; Erdaw et al., 2017b) and reduce various proteinaceous antinutrient factors associated with the SBM diet (Rooke et al., 1998). Our results indicated that protease supplementation increased the coefficient of apparent ileal digestibility of essential AA like Lys, Thr, Trp, Val, and CP in 90 and 95% dAA/CP level diets. These results are consistent with Vieira et al. (2013) and Freitas et al. (2011), who observed that the inclusion of exogenous protease improved the apparent ileal digestibility of CP, Lys, Thr, and TSAA. Exogenous protease works by releasing peptides from antinutritional components in feed ingredients, cleaving links between AA and protein complexes (López-Otín and Bond, 2008), supplementing endogenous peptidase activity, and lowering enzymatic secretion and protein turnover, resulting in AA for protein synthesis and deposition (Freitas et al., 2011). Protease supplementation in the diet can enhance endogenous trypsin activity and also increase the villus height: crypt depth ratio in the duodenum and jejunum (Ding et al., 2016). A study also reported that supplementation of protease and phytase together improves the effect of both enzymes compared to when used alone Vieira et al. (2013). Phytase can complement protease activity because crops have spherical phytate molecules, usually stored in protein-rich tissues such as the germ and aleurone layer. Protein and phytate solubilities are very similar, resulting in strong chelation between globules of phytate molecules and protein in crops (Jiang et al., 2020). Therefore, the addition of phytase could prevent the excessive formation of protein-phytate chelates (Boling-Frankenbach et al., 2001). This is the possible reason for the increase in apparent ileal digestibility of various AA in our experiment. Furthermore, the pattern of increase in digestibility shown here is comparable to that previously described (Angel et al., 2011; Cowieson et al., 2018), in which the increase in AA digestibility was notably substantial for Thr, Lys, Val, and Cys rather than for Met at 90 and 95% CP level (Angel et al., 2011) and 97.5, 95, and 92.5% CP level (Cowieson et al., 2018). These responses are linked to the intrinsic digestibility of specific amino acids as well as the amino acid composition of endogenous proteins. Therefore, it can be inferred that protease effects are more pronounced when the diets are formulated to digestible AA. As shown by Wang et al. (2020b), protease supplementation increased the standardized apparent ileal digestibility of Thr, Phe, and His in 13.5% as compared to 17.5% CP in diet. This finding offers the intriguing concept that low AA/CP level diets benefit from exogenous protease supplementation with improved amino acid digestibility. Furthermore, Wang et al. (2020a) showed that reducing the CP levels in the diet from 17.5% to 15.5% and then to 13.5% damaged the intestinal integrity of Peking ducks. However, the inclusion of protease could partly attenuate these negative effects by significantly increasing serum-free glutamic acid concentration and decreasing plasma endotoxins, IL-6, and cecal isovalerate concentration, but these physiological benefits might not be enough to positively affect the productive performance. Also, similar findings were reported by Freitas et al. (2011), who found that protease supplementation does not affect the growth performance but did find a substantial improvement in apparent amino acid, nitrogen, and energy digestibility in broilers. Similar results were found by Angel et al. (2011), where protease supplementation did not affect broiler performance characteristics but showed an improvement in the digestibility of CP, Arg, Ile, Lys, Thr, Asp, His, Cys, and Ser.

Proteases increase the digestibility of proteins and amino acids, especially when the ingredients have low quality or low bioavailability (Kocher et al., 2002). Thus, besides providing nutritional benefits, enzymes can contribute to maintaining the normal balance of the host microbiome, reflecting better conditions for performance and egg production (Vieira Filho et al., 2015, Chen et al., 2021). Therefore, including protease is beneficial, especially in low crude protein diets. One of the limitations of our research is that we did not perform precise feeding on our birds. Nevertheless, the feed consumption is similar in all treatment groups indicating that lower AA/CP level groups compensated for lower protein by decreasing egg weight and egg production. In the future, it would be interesting to see the effects of protease supplementation in diets with animal protein, DDGS, and other less digestible protein sources (canola meal, sunflower meal).

Feed cost is a substantial recurring cost of egg production. There were differences in egg production and egg quality with decreasing at 85% dAA/CP level, unsurprisingly, it also had a negative effect on the egg income, feed cost, and return on investment. Feed cost increased with increasing dAA/CP level, as predicted, but the higher feed cost of the 100% treatment did not result in numerically higher egg revenue. Hence the return over feed cost for the 100% dAA/CP level was not advantageous. These findings show that, while hens fed protein-dense diets had better feed efficiency and produced more eggs of greater sizes, the egg income from these activities did not cover the higher costs of the high protein-dense diets. These results are similar to dePersio et al. (2015), who observed that profit increased linearly with increasing energy and nutrient density from 85, 90, 95, 100, and 105% in Hy-Line W-36 from 19 to 70 wk of age. Egg income and feed cost per hen grew as energy and nutrient density increased, but profit decreased. Similar to our results, dePersio et al. (2015) reported that increasing the energy and nutritional density of Hy-Line W-36 increases egg production, egg weight, egg mass, feed efficiency, energy intake, BW, egg income, and feed cost but decreases profit. These conclusions from previous research and the one from this experiment can be extrapolated to commercial egg producers.

Current research results showed that a decrease in dietary dAA/CP level at 85% leads to a reduction in egg production, egg mass, feed conversion ratio, and egg income. The inclusion of protease reduced the overall feed intake and FCR-D, in dAA/CP level deficient diets (90% and 95% CP levels) and improved the digestibility of Lys, Thr, Trp, and Val.
